# Higher Levels of Lipoprotein Associated Phospholipase A2 is associated with Increased Prevalence of Cognitive Impairment: the APAC Study

**DOI:** 10.1038/srep33073

**Published:** 2016-09-09

**Authors:** Ruixuan Jiang, Shengyun Chen, Yuan Shen, Jianwei Wu, Shuohua Chen, Anxin Wang, Shouling Wu, Xingquan Zhao

**Affiliations:** 1 Department of Neurology, Beijing Tian Tan Hospital, Capital Medical University, Beijing, 100050, China; 2 China National Clinical Research Center for Neurological Diseases, Beijing, 100050, China; 3 Center of Stroke, Beijing Institute for Brain Disorders, Beijing, 100050, China; 4 Beijing Key Laboratory of Translational Medicine for Cerebrovascular Disease, Beijing, 100050, China; 5 Department of Cardiology, Kailuan Hospital, North China University of Science and Technology, Tangshan, 063000, China; 6 Department of Epidemiology and Health Statistics, School of Public Health, Capital Medical University, Beijing, 100050, China

## Abstract

Lipoprotein-associated phospholipase A_2_ (Lp-PLA_2_) is a unique circulating phospholipase with inflammatory and oxidative activities and the limited data regarding the relationship between Lp-PLA_2_ and cognitive impairment are conflicted. We conducted a cross-sectional study including 1,374 Chinese adults recruited from 2010 to 2011, aiming to evaluate the relationship between Lp-PLA_2_ levels and the prevalence of cognitive impairment in a Chinese community-based population. Participants underwent standardized evaluation. Serum Lp-PLA2 mass was measured by ELISA. Cognition status was evaluated via the Mini-Mental Status Exam (MMSE) and cognitive impairment was identified as MMSE <24. Multivariable logistic regression models were used to assess the associations of Lp-PLA_2_ mass with cognitive impairment. Lp-PLA_2_ mass was significantly associated with the prevalence of cognitive impairment after adjusting for other potential confounding factors (compared with the first quartile, adjusted ORs of the second, third, and fourth quartile were 2.058 (95% CI, 0.876–4.835), 2.834 (95% CI, 1.255–6.398), and 4.882 (95% CI, 2.212–10.777), p < 0.0001). In conclusion, elevated level of Lp-PLA_2_ mass was independently associated with the prevalence of cognitive impairment in Chinese adults.

Cognitive impairment has become a burgeoning public health problem and is predicted to increase dramatically worldwide in the coming decades due to ageing population. This brings a compelling need for the discovery, validation, and standardization of screening instruments that can be used in diagnosis of pre-clinical and symptomatic stages of the disease.

Lipoprotein-associated phospholipase A_2_ (Lp-PLA_2_), also known as platelet activating factor acetylhydrolase (PAF-AH), is a unique circulating phospholipase that primarily bound to the low-density lipoprotein (approximately 80%) with inflammatory and oxidative activities associated with cardiovascular disease and cerebrovascular events independent of traditional risk factors^1^,^2^,^3^. Since cardiovascular and cerebrovascular risk factors may increase the risk of cognitive impairment, Lp-PLA_2_ may also be related with cognitive impairment. The limited data regarding the relationship between Lp-PLA_2_ mass or activity and cognitive impairment is conflicted^4^,^5^,^6^.

The objective of the current study was to evaluate the relationship of serum Lp-PLA_2_ mass with the prevalence of cognitive impairment in a Chinese community-based population. We hypothesized that the population with higher levels of Lp-PLA_2_ mass would have a higher prevalence of cognitive impairment.

## Results

### Characteristics and Risk Factors Associated with Cognitive Impairment

A total of 1,374 subjects, 71.8% men and 28.2% women, were analyzed. The baseline characteristics of participants according to quartiles of Lp-PLA_2_ mass are presented in Table 1. The mean value of Lp-PLA_2_ mass from the lowest quartile to the highest quartile was 127.66 ± 2.67 ng/ml, 135.58 ± 2.45 ng/ml, 147.06 ± 4.71 ng/ml, and 253.50 ± 202.31 ng/ml, respectively. We found that there were significant differences in age, body mass index, low-density lipoprotein cholesterol (LDL), high-density lipoprotein cholesterol (HDL), triglycerides, white blood cell count, alanine transaminase, and homocysteine among the different quartiles. These variables, with an exception of age and HDL, tended to be decreased in the higher Lp-PLA_2_ quartile groups, whereas age and HDL levels were higher in the higher Lp-PLA_2_ quartile groups. Also, there were significant differences in the proportion of participants who have working environment with dust, cigarette smoking, past history of hyperlipidemia, hypertension, anti-hypertensive drug use, anti-diabetic drug use among the different quartiles.

### Higher Lp-PLA_2_ mass is associated with the increased prevalence of cognitive impairment independent of other potential confounding factors

**Figure 1:**
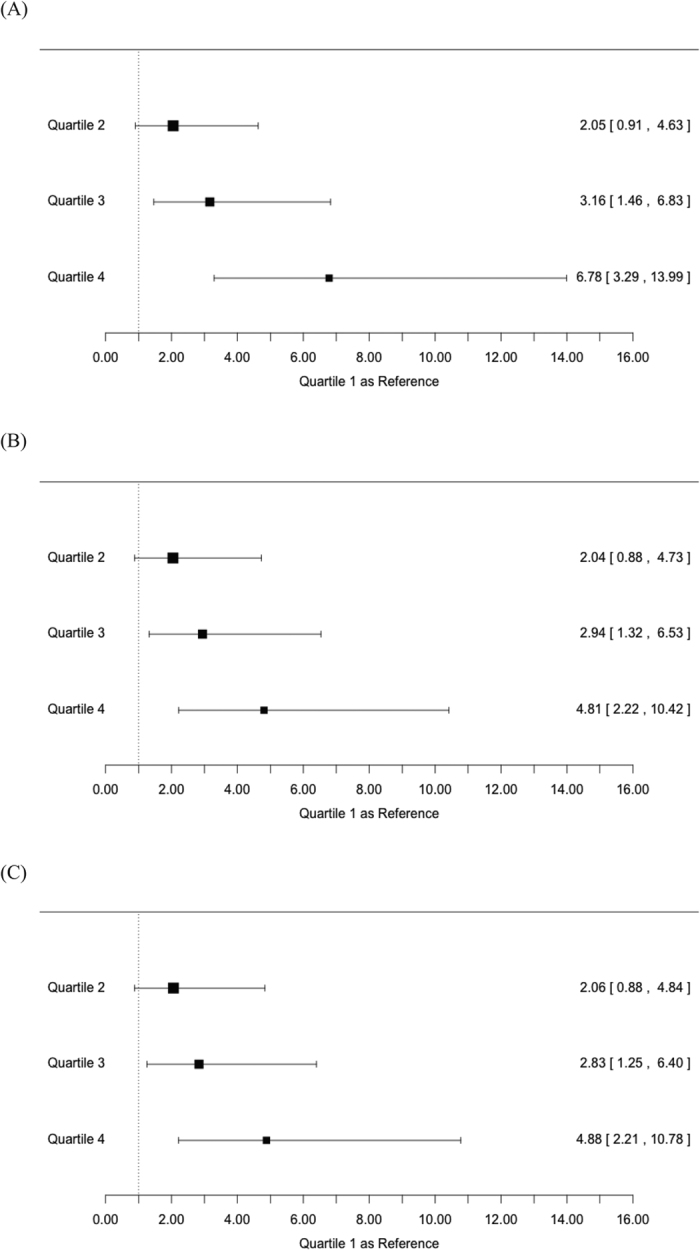
Odd ratios with 95% confidence interval for cognitive impairment (MMSE < 24) according to baseline Lp-PLA2 quartiles: (**A**) crude OR; (**B**) OR adjusted for age and sex; (**C**) OR adjusted for age, sex, education, body mass index, low-density lipoprotein cholesterol, high-density lipoprotein cholesterol, triglycerides, total cholesterol, fasting blood glucose, white blood cells counts, current smoking, alcohol intake, hyperlipidemia, lipid lowering drugs, hypertension, diabetes, myocardial infarction, and physical inactivity.

Cognitive impairment, defined as MMSE scored <24 points, were identified in 107 participants of the total 1,374 participants. Cognitive impairment prevalence from the lowest quartile to the highest quartile of Lp-PLA_2_ mass were 2.6%, 5.2%, 7.8% and 15.5%, respectively (p < 0.0001). Compared with the first quartile, age-, sex- and education adjusted odd ratios (OR) for the second, third, and fourth quartile were 2.044 (95% CI, 0.883–4.731), 2.940 (95% CI, 1.323–6.534), and 4.808 (95% CI, 2.219–10.420), respectively, p value for trend <0.0001 (Table 2). Additional adjustments for age, sex, education, working environment with dust, current smoking, current alcohol use, hyperlipidemia, lipid-lowering drugs use, hypertension, anti-hypertensive drugs use, diabetes, anti-diabetic drugs use, physical inactivity, low-density lipoprotein cholesterol, high-density lipoprotein cholesterol, triglycerides, total cholesterol, body mass index, fasting blood glucose, alanine transaminase, and C-reactive protein did not affect the significance, with ORs for the second, third, and fourth quartile 2.058 (95% CI, 0.876–4.835), 2.834 (95% CI, 1.255–6.398), and 4.882 (95% CI, 2.212–10.777), respectively, p value for trend <0.0001. The increased levels of Lp-PLA_2_ mass were associated with an increased prevalence of cognitive impairment (Fig. 1).

## Discussion

In our study, the higher levels of Lp-PLA_2_ mass were associated with increased prevalence of cognitive impairment. There was a significant trend over the quartiles and a compelling gradient of increased prevalence of cognitive impairment for levels of Lp-PLA_2_. The association was independent of other cardiovascular risk factors and could not be explained by other risk factors associated with cognitive impairment.

Previous studies have evaluated the association between Lp-PLA_2_ activity or mass and cognitive impairment. Agreement among researchers is not univocal and the possible pathological process is still unclear. Because of the conflicted outcomes of these studies, more data was essential in order to obtain information on the association.

In the Rotterdam Study^7^,^8^, individuals with increasing levels of Lp-PLA_2_ were associated with an increased risk of developing cognitive impairment over a mean follow-up of 5.7 years and people in the upper quartile had a 56% higher risk compared with those in the lower quartile. This association of Lp-PLA_2_ appeared stronger with vascular dementia. Similar results were repeated in an analysis of 3,320 participants of community-dwelling adults age ≥65 years in the Cardiovascular Health Study (CHS)^5^ followed for an average of 5.4 years showed that with each standard deviation higher Lp-PLA_2_ mass and activity were related to increased risk of cognitive impairment. Participants in the highest quartile of Lp-PLA_2_ mass were 50% more likely to develop cognitive impairment than those in the lowest quartile in adjusted models. Lp-PLA_2_ activity also doubled the risk of mixed dementia in the highest compared to lowest quartile. In addition, a recent case-control study showed that subjects with higher levels of Lp-PLA_2_ were almost twice as likely to have Alzheimer’s disease (AD) compared with subjects with Lp-PLA2 levels below the median^9^. Our results supported the idea that higher Lp-PLA_2_ is associated with increased prevalence of cognitive impairment. The associations found here may support the potential value of Lp-PLA_2_ in the development and treatment of cognitive decline.

However, the analysis from the Framingham Study^6^ failed to replicate these association as Lp-PLA_2_ mass was not found to be associated with an increased risk of dementia or Alzheimer’s disease. The authors proposed that this lack of association could be because of a relatively small number of incident AD cases in the sample and that the participants with dementia were more severe and deceased at follow-up. Also, the lower level of inflammation in Framingham study participants may have been a factor in the lack of associations should there be a threshold in Lp-PLA_2_ mass for detecting associations with dementia. Another cross-sectional study^10^ also found no significant difference in the Lp-PLA_2_ activity between cognitive impairment group and controls. Though the mean Lp-PLA_2_ activity observed in their control group (195.4 nmol/min/ml, SD 41.9) was higher than anticipated, based on levels observed in the Framingham Offspring study (144 nmol/min/ml, SD 36)^11^ and the Dallas Heart Study (146 nmol/min/ml, SD 40)^12^. Possibly a threshold effect is present, although a standardized cut-point for Lp-PLA_2_ levels is still unclear.

Elevated levels of inflammatory biomarkers such as IL-6 and C-reactive protein were found in plasma of the patients with AD years before the clinical dementia syndrome developed^13^,^14^. Besides a few studies^15^,^16^, most of the evidence supports inflammation as an important factor in the development of cognitive impairment and supports the idea that inflammatory biomarkers might be useful to help identify the people at higher risk of cognitive impairment^17^. Evidence of an association between Lp-PLA_2_ and cognitive impairment would back up the hypothesis that inflammation is involved in the pathogenesis of cognitive deficit and decline. Lp-PLA_2_ may act in the pathogenesis of cognitive impairment by directly and independently affecting the brain or key molecules that are implicated in dementia such as amyloid and tau^18^. Another possible way may be by increasing vascular damage and promoting neural degeneration with loss of cognitive reserve^19^. For its pro-inflammatory role and close association with lipoproteins has been shown to be associated with the presence of advanced lesions leading to plaque instability and clinical events^7^,^20^,^21^. Also, the levels of oxidized LDL in AD patients were significantly higher compared with controls and it is also increased with severity of disease^22^. As Lp-PLA_2_ hydrolyzes oxidized low-density lipoproteins producing lysophosphatidylcholine (LysoPC) and oxidized nonesterified fatty acids (OxNEFA)^3^,^21^ and the brain is believed to be especially vulnerable to oxidative stress as it contains high concentrations of fatty acids that are susceptible to lipid peroxidation. It is possible that Lp-PLA_2_ exhibits a dual action which might provide an opportunity for manipulation of Lp-PLA_2_ modulation, such as darapladib^23^, an inhibitor of Lp-PLA_2_. In addition, a chronic, low-level inflammation can occur throughout the body in response to various factors, among them a poor diet or exposure to mold and toxins. Nevertheless, some dietary changes, such as including “healthy fats” such as omega-3, may offset the damage from oxidative stress and inflammation and increase chances of maintaining a healthy brain^24^. Whether there is any correlation between increased Lp-PLA_2_ levels and severity of cognitive impairment can also be further explored to better explain the association. However, this was not feasible in the current study due to the small numbers in each of the severity categories of cognitive impairment. Such relation and its underlying mechanism need to be further studied. To establish the mechanistic links between a single risk factor such as inflammation marker (e.g. Lp-PLA_2_) and cognitive impairment is difficult. The development of cognitive impairment is complex and complicated by the fact that several diseases often co-exist and share some common risk factors including Lp-PLA_2_. A better approach to mechanism understanding could be formulating the hypothesis on a “metabolic-cognitive syndrome” to explain the complex relationship between metabolic disorders (i.e. diabetes, hypertension, atherosclerosis, inflammation and other risk factors) and cognitive disturbances and the boundaries between normal and pathological condition^25^.

To our knowledge, no published articles have assessed this association in Chinese population to date. As cognitive impairment is a major public health problem worldwide, especially to the growing aging population in China, understanding the role of Lp-PLA_2_ in the development of cognitive impairment may be helpful for the prevention of this disease and its consequences. However, the following limitations should be noted. First, the MMSE was employed to assess cognitive status in this study and this score does not always reflect the cognitive function exactly, as it is sometimes influenced by the subjects’ education level^26^. Although a multitude of potential confounders including education level were considered to minimize their impacts. Ceiling effects may also limit the usefulness of MMSE in detecting cognitive impairment. The MMSE examinations were done during follow-up visits in 2012, which may not exactly reflect the participants’ mental status at the time of blood sampling (in 2010). However, since we excluded participants with stroke history as well as other major diseases and cognitive impairment is a chronic process, so the follow-up MMSE were used to represent the cognitive status at baseline. Secondly, our study was based on a subgroup of participants of the APAC Study. Compared with those excluded from the analyses, the included participants were older (study population 59.09 ± 10.88 versus excluded 53.39 ± 11.51) and had more males (71.8% male in the study population versus 55.3% in the excluded population). The proportion of participants with hypertension (study population 57.1% versus excluded 44.0%) and diabetes (study population 15.7% versus excluded 10.3%) were also different. So the findings might not be generalizable to other populations. We cannot totally exclude the possibility of selection bias in that relative healthy individuals may be more likely to refuse to participate in the free medication examination. It should also be noted that clinical conditions (e.g. liver dysfunction), drugs (e.g. antiplatelet/antithrombotic drugs) and genotype may alter the Lp-PLA_2_ production and activity^3^. Participants with cerebral infarction or coronary artery diseases were excluded which would be the major reason for assumption of antiplatelet/antithrombotic drugs. We did not evaluate genotype associations in the current study, which may be included in future studies. Also, studies have shown that dietary changes may contribute to the prevention of cognitive impairment. However, we did not have detailed information concerning dietary habits in our study. More variables concerning dietary, lifestyle and genotype may be added to further study these effects. Furthermore, the present study is cross-sectional in design so these results need to be further investigated in a longitudinal design to allow conclusions regarding causality. As the study is ongoing, future analyses will allow us to further explore this association.

In conclusion, elevation of Lp-PLA_2_ mass levels was significantly associated with higher prevalence of cognitive impairment independent of several vascular, inflammatory and other risk factors.

## Methods

### Study Design and Population

The APAC (Asymptomatic Polyvascular Abnormalities in Community) Study is a community-based, ongoing observational study that aims to investigate the asymptomatic poly-vascular abnormalities in Chinese adults^27^,^28^. This community-based study cohort was derived from a previously described population of the Kailuan Study^27^, which included a total of 101,510 participants (81,110 men) of the Kailuan community, in Tangshan city, a large modern city Southeast of Beijing. From June 2010 to June 2011, a sample of 7000 subjects older than 40 years was randomly selected from the Kailuan cohort, using a stratified random sampling method by age and gender based on the data of the Chinese National Census from 2010. The sample size was calculated based on detection of 7% of the event rate with 0.7% precision (α = 0.05). The response rate was assumed to be >80%. A total of 5,852 subjects signed consent to participate in the APAC study and 5,816 people eventually completed the baseline data collection. Among these 5,816 individuals, 376 subjects were not included because of not meeting the inclusion criteria as following: (1) no history of stroke, transient ischemic attack, and coronary disease at baseline as assessed by a validated questionnaire; and (2) absence of neurologic deficits indicating previous stroke as examined by experienced physicians. At last, a total of 5,440 participants were included in the APAC Study. The detailed recruitment protocol has previously been reported elsewhere^29^.

**Figure 2:**
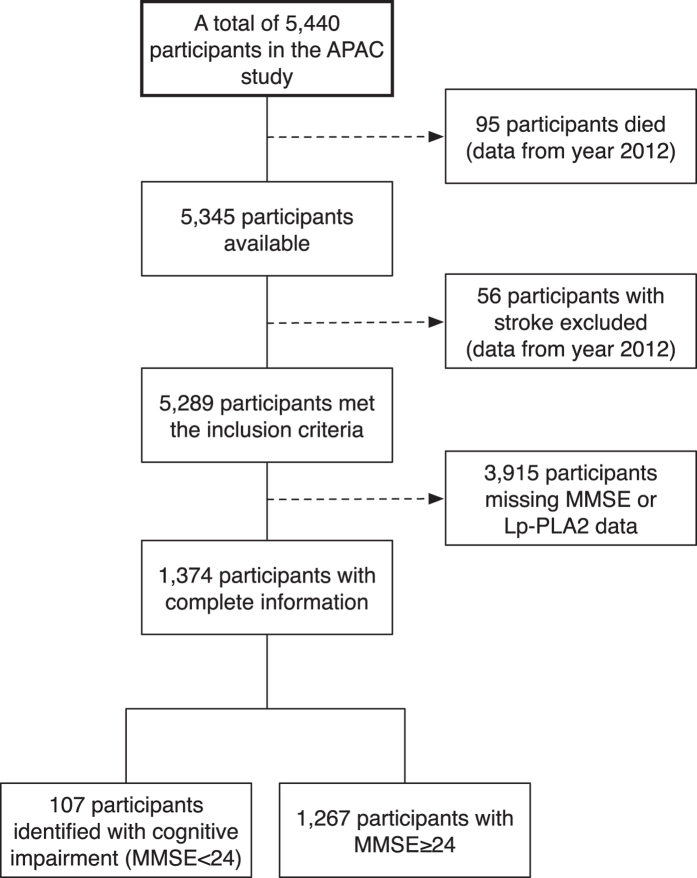
Flowchart of the study.

This is a further and in-depth analysis of the APAC study. We excluded 151 participants who died or had a stroke from recruitment to 2012, and 3,915 participants who had incomplete data on MMSE or Lp-PLA_2_ data, leaving 987 men and 387 women (Fig. 2) available for the analyses. All participants provided written informed consent and informed of abnormal findings and recommended treatments. The study protocol conformed to the ethical guidelines of the 1975 Declaration of Helsinki. The study was also approved by the Ethics Committee of the Kailuan General Hospital and Beijing Tiantan Hospital.

### Measurement of Lp-PLA_2_

Blood samples were collected from the antecubital veins of participants before 9:00 AM after an overnight fast. Each sample was collected into two tubes, EDTA (Ethylene Diamine Tetraacetic Acid) tube (to obtain plasma) and serum separator tube (to obtain serum). The samples were centrifuged 10 min by 3000 r/min (serum separator tube was to clot for two hours at room temperature before centrifuge), plasma and serum were both collected in separate EP tubes. Samples were frozen as rapidly as possible to −80 °C for storage until laboratory determinations were performed. Lp-PLA_2_ concentration in the serum (mass) was measured using enzyme linked immunosorbent assay (CUSABIO company, Human lipoprotein phospholipase A2 (Lp-PLA_2_) enzyme immunoassay kit, Catalog number CSB-E08319h). The intra-assay and inter-assay coefficients of variation are <8% and <10%. In order to reduce inter-assay error and measurement error, we assessed the serum levels of Lp-PLA_2_ mass by professional technicians using enzyme linked immunosorbent assay (CUSABIO company, Human lipoprotein phospholipase A2 (Lp-PLA_2_) enzyme immunoassay kit, Catalog number CSB-E08319h) at the same time in Beijing Tiantan Hospital affiliated to Capital Medical University, Beijing, China. Experimental procedures and results of the judging were made according to the kit instructions strictly.

### Ascertainment of Cognitive Impairment

The Mini-Mental Status Exam (MMSE)^30^ was used as a cognitive screener which contain 18 items that assesses orientation, memory, attention, ability to follow commands, and copying a geometric figure. Scores range from 0 to 30 points, with a lower score reflecting greater cognitive impairment. Specially trained physicians or assistants tested all 1,374 participants with the MMSE during their follow-up visit to the research hospital in 2012. Previous studies using MMSE as a screening tool for dementia recommended a cut-off point of score <24 on the MMSE as suspect of cognitive impairment^30^,^31^,^32^,^33^. In our study, we defined participants with a MMSE <24 as subjects with cognitive impairment and MMSE ≥24 as absent of cognitive impairment.

### Assessment of demographic variables and potential covariates

A standardized questionnaire was used for collecting information on subjects’ demographic and socioeconomic data, family and past history. Attained level of education was grouped into those with at most primary education, those with junior education or vocational training, and those with senior vocational or academic education.

All subjects were measured by standing in light clothing without shoes and hats. Height was measured to the nearest 0.1 cm using a portable stadiometer and weight was measured to the nearest 0.1 kg using calibrated platform scales. Then, body mass index (BMI) was calculated as weight (kg)/height (m)^2^.

Information on smoking, alcohol intake and physical activity and past history was collected via questionnaires. History of diseases mainly included hypertension, diabetes mellitus, and hyperlipidemia. Hypertension was defined as presence of a history of hypertension, or using antihypertensive medication, or a SBP ≥140 mm Hg, or a DBP ≥90 mm Hg. Diabetes mellitus was defined as a self-reported history, currently treated with insulin or oral hypoglycemic agents, or fasting blood glucose level ≥7.0 mmol/L (126 mg/dl). Dyslipidemia was defined as a self-reported history, current use of cholesterol lowering medicine, or total cholesterol level ≥5.7 mmol/L (220 mg/dl) or triglyceride ≥1.7 mmol/L (150 mg/dl) or low-density lipoprotein cholesterol (LDL-C) ≥130 mg/dl.

Methods for collecting the fasting blood specimens, measuring glucose and lipids, and the definitions of other vascular risk factor covariates in the APAC study have been described previously^34^,^35^.

### Statistical Analysis

The participants were classified into 4 groups according to the quartile of Lp-PLA_2_ mass. In addition, medians and proportions of potential risk factors for cognitive impairment among these 4 groups were calculated. Continuous variables were presented as mean ± standard deviation and compared using Kraskal-Wallis test. Categorical variables were presented as frequencies (percentage) and compared using the Chi-squared test. Logistic regression was used to evaluate the relationship between Lp-PLA_2_ mass and cognitive impairment (MMSE < 24) by calculating the odds ratio (OR) and 95% confidence interval (CI). The possible confounders such as age, sex, education, working environment with dust, current smoking, current alcohol use, hyperlipidemia, lipid-lowering drugs use, hypertension, anti-hypertensive drugs use, diabetes, anti-diabetic drugs use, physical inactivity, low-density lipoprotein cholesterol, high-density lipoprotein cholesterol, triglycerides, total cholesterol, body mass index, fasting blood glucose, alanine transaminase, and C-reactive protein were adjusted in the statistical analysis. Trend test was performed to examine whether there was a dose-response relationship between Lp-PLA_2_ levels and cognitive impairment. Statistical analysis was performed using SAS software, version 9.3 (SAS Institute Inc., Cary, NC, USA). All CIs were estimated at the 95% level and significance was set at a P value of <0.05 (2-sided).

## Additional Information

**How to cite this article**: Jiang, R. *et al*. Higher Levels of Lipoprotein Associated Phospholipase A2 is associated with Increased Prevalence of Cognitive Impairment: the APAC Study. *Sci. Rep.* **6**, 33073; doi: 10.1038/srep33073 (2016).

## Figures and Tables

**Table 1:** Baseline characteristics of participants according to Lp-PLA2 quartiles.

	Total	Lp-PLA2 quartiles				
		Q1	Q2	Q3	Q4	P value
Number of subjects		343	344	344	343	
MMSE <24, n (%)	107 (7.8)	9 (2.6)	18 (5.2)	27 (7.8)	53 (15.5)	<0.0001
Lp-PLA2 mass, ng/ml	165.91 ± 113.16	127.66 ± 2.67	135.58 ± 2.45	147.06 ± 4.71	253.50 ± 202.31	<0.0001
Age, years	59.09 ± 10.88	55.26 ± 8.47	57.47 ± 9.74	59.87 ± 10.61	63.75 ± 12.48	<0.0001
Male, n (%)	987 (71.8)	254 (74.1)	257 (74.7)	237 (68.9)	239 (69.7)	0.2127
Education, n (%)						
Illiteracy or primary school	223 (16.2)	50 (14.6)	48 (14.0)	53 (15.4)	72 (21.0)	0.1485
Middle school	599 (43.6)	160 (46.7)	153 (44.5)	144 (41.9)	142 (41.4)	
High school or higher	552 (40.2)	133 (38.8)	143 (41.6)	147 (42.7)	129 (37.6)	
Working environment with dust, n (%)	339 (24.7)	106 (30.9)	74 (21.5)	86 (25.0)	73 (21.28)	0.0109
Current smoking, n (%)	534 (38.9)	158 (46.1)	146 (42.4)	113 (32.9)	117 (34.1)	0.0005
Current alcohol use, n (%)	537 (39.1)	150 (43.7)	143 (41.6)	123 (35.8)	121 (35.3)	0.0528
Hyperlipidemia, n (%)	679 (49.4)	186 (54.2)	177 (51.5)	168 (48.8)	148 (43.2)	0.027
Lipid-lowering drugs use, n (%)	23 (1.7)	8 (2.3)	5 (1.5)	3 (0.9)	7 (2.0)	0.4524
Hypertension, n (%)	785 (57.1)	178 (51.9)	179 (52.0)	219 (63.7)	209 (60.9)	0.0014
Anti-hypertensive drugs use, n (%)	356 (25.9)	68 (19.8)	74 (21.5)	114 (33.1)	100 (29.2)	<0.0001
Diabetes, n (%)	216 (15.7)	47 (13.7)	55 (16.0)	64 (18.6)	50 (14.6)	0.3117
Anti-diabetic drugs use, n (%)	124 (9.0)	23 (6.7)	25 (7.3)	47 (13.7)	29 (8.5)	0.0054
Physical inactivity, n (%)	493 (35.9)	129 (37.6)	114 (33.1)	114 (33.1)	136 (39.6)	0.1862
BMI, kg/m^2^	24.97 ± 3.15	25.41 ± 2.89	25.13 ± 3.19	24.98 ± 3.08	24.35 ± 3.33	<0.0001
LDL, mmol/L	2.68 ± 0.82	2.76 ± 0.84	2.70 ± 0.73	2.64 ± 0.71	2.61 ± 0.96	0.0432
HDL, mmol/L	1.62 ± 0.45	1.60 ± 0.44	1.56 ± 0.44	1.61 ± 0.41	1.69 ± 0.48	0.0057
TG, mmol/L	1.69 ± 1.42	1.87 ± 1.59	1.79 ± 1.41	1.62 ± 1.41	1.47 ± 1.23	<0.0001
TC, mmol/L	5.20 ± 1.06	5.29 ± 1.25	5.23 ± 0.99	5.15 ± 0.96	5.16 ± 1.02	0.7490
FBG, mmol/L	5.70 ± 1.61	5.68 ± 1.50	5.75 ± 1.65	5.81 ± 1.83	5.54 ± 1.45	0.0751
White cell count, × 109/L	6.38 ± 1.63	6.49 ± 1.58	6.45 ± 1.72	6.40 ± 1.58	6.16 ± 1.63	0.0293
ALT, U/L	18.58 ± 14.71	19.10 ± 12.17	18.70 ± 11.75	20.26 ± 21.72	16.26 ± 10.04	<0.0001
CRP, mg/L	2.17 ± 3.19	1.94 ± 2.66	1.98 ± 2.50	2.19 ± 3.11	2.57 ± 4.18	0.5317
Homocysteine, umol/l	17.04 ± 9.44	16.81 ± 10.30	16.71 ± 9.61	15.39 ± 7.90	19.25 ± 9.43	<0.0001

Data are expressed as mean ± SD or n (%).

Abbreviation: MMSE = Mini-Mental Status Exam; LDL = low-density lipoprotein cholesterol; HDL = high-density lipoprotein cholesterol; TG = triglycerides; TC = total cholesterol; BMI = body mass index; FBG = fasting blood glucose; ALT = alanine transaminase; CRP = C-reactive protein.

**Table 2:** Odd ratios (95% CI) for cognitive impairment (MMSE <24) according to baseline Lp-PLA2 quartiles.

	Quartiles 1	Quartiles 2	Quartiles 3	Quartiles 4	p for trend
Crude	Reference	2.049 (0.907–4.627)	3.161 (1.464–6.826)	6.782 (3.288–13.989)	<0.0001
Model 1^1^	Reference	2.044 (0.883–4.731)	2.940 (1.323–6.534)	4.808 (2.219–10.420)	<0.0001
Model 2^2^	Reference	2.058 (0.876–4.835)	2.834 (1.255–6.398)	4.882 (2.212–10.777)	<0.0001

Data expressed by odds ratio (95% CI, confidence interval) and p value.

^1^Adjusted for age, sex and education.

^2^Adjusted for age, sex, education, working environment with dust, current smoking, current alcohol use, hyperlipidemia, lipid-lowering drugs use, hypertension, anti-hypertensive drugs use, diabetes, anti-diabetic drugs use, physical inactivity, low-density lipoprotein cholesterol, high-density lipoprotein cholesterol, triglycerides, total cholesterol, body mass index, fasting blood glucose, alanine transaminase, and C-reactive protein.
